# Does Prefrontal Glutamate Index Cognitive Changes in Parkinson’s Disease?

**DOI:** 10.3389/fnhum.2022.809905

**Published:** 2022-04-12

**Authors:** Isabelle Buard, Natalie Lopez-Esquibel, Finnuella J. Carey, Mark S. Brown, Luis D. Medina, Eugene Kronberg, Christine S. Martin, Sarah Rogers, Samantha K. Holden, Michael R. Greher, Benzi M. Kluger

**Affiliations:** ^1^Department of Neurology, University of Colorado, Denver, Aurora, CO, United States; ^2^Department of Medicine, University of Wisconsin–Madison, Madison, WI, United States; ^3^Department of Psychiatry, University of Colorado, Denver, Aurora, CO, United States; ^4^Department of Psychology, University of Houston, Houston, TX, United States; ^5^Department of Neurosurgery, University of Colorado, Denver, Aurora, CO, United States; ^6^Department of Neurology, University of Rochester Medical Center, Rochester, NY, United States

**Keywords:** Parkinson’s disease, mild cognitive impairment, magnetic resonance spectroscopy, dorsolateral prefrontal cortex, glutamate

## Abstract

**Introduction:**

Cognitive impairment is a highly prevalent non-motor feature of Parkinson’s disease (PD). A better understanding of the underlying pathophysiology may help in identifying therapeutic targets to prevent or treat dementia. This study sought to identify metabolic alterations in the prefrontal cortex (PFC), a key region for cognitive functioning that has been implicated in cognitive dysfunction in PD.

**Methods:**

Proton Magnetic Resonance Spectroscopy was used to investigate metabolic changes in the PFC of a cohort of cognitively normal individuals without PD (CTL), as well as PD participants with either normal cognition (PD-NC), mild cognitive impairment (PD-MCI), or dementia (PDD). Ratios to Creatine (Cre) resonance were obtained for glutamate (Glu), glutamine and glutamate combined (Glx), N-acetylaspartate (NAA), myoinositol (mI), and total choline (Cho), and correlated with cognitive scores across multiple domains (executive function, learning and memory, language, attention, visuospatial function, and global cognition) administered to the PD participants only.

**Results:**

When individuals retain cognitive capabilities, the presence of Parkinson’s disease does not create metabolic disturbances in the PFC. However, when cognitive symptoms are present, PFC Glu/Cre ratios decrease with significant differences between the PD-NC and PPD groups. In addition, Glu/Cre ratios and memory scores were marginally associated, but not after Bonferroni correction.

**Conclusion:**

These preliminary findings indicate that fluctuations in prefrontal glutamate may constitute a biomarker for the progression of cognitive impairments in PD. We caution for larger MRS investigations of carefully defined PD groups.

## Highlights

- Prefrontal alterations in PD may be relevant to cognitive decline
- Decreased prefrontal glutamate is observed in PD-related cognitive dysfunction
- Prefrontal glutamate could be a biomarker of cognitive changes in PD

## Introduction

In addition to motor features, cognitive decline affects a large proportion of Parkinson’s disease (PD) patients (Saredakis et al., 2019). PD with mild cognitive impairment (PD-MCI) is a transitional state between normal cognition “PD-NC” and Parkinson’s disease dementia “PDD” (Litvan et al., 2012), characterized by increased difficulty in performing executive function, verbal memory, and object recognition tasks. PD-MCI is strongly associated with conversion to PDD (Nicoletti et al., 2019), often resulting in poor patient and caregiver quality of life (Obeso et al., 2017), and nursing home placement (Buchanan et al., 2002).

Cognitive impairment in PD is associated with disturbances in frontostriatal circuits influenced by dopaminergic abnormalities (Leh et al., 2010). However, glutamatergic abnormalities are also observed in frontal areas, such as changes in glutamatergic transporters homeostasis (Kashani et al., 2007), but focal metabolic changes may only be relevant to specific functions. Glutamatergic pathways are therefore suspected to play a major role in the structural and functional organization of these circuits (Albin et al., 1989) and possibly in cognitive changes in PD. Functional and connectivity alterations in the prefrontal cortex (PFC) are specifically associated with PD-related impairment in executive function (Isaacs et al., 2019). However, people with PD experience difficulties in other cognitive domains (Watson and Leverenz, 2010) including memory, language, attention, and visuospatial abilities. Both anatomical and functional neuroimaging provide important insights into prefrontal abnormalities associated with cognitive decline in PD.

Proton magnetic resonance spectroscopy (^1^H-MRS) identifies focal metabolic and neurodegenerative changes (Martin, 2007). Relevant metabolites that can be accessed include: N acetyl-aspartate (NAA), a putative marker of neuronal viability and function; creatine, a marker of energy reserves in the tissue; myo-Inositol (mI), a glial marker (Oz et al., 2014), choline and choline containing compounds (Cho), an indicator of glial health and also inflammation (Rovira and Alonso, 2013); and glutamate, the primary excitatory neurotransmitter in the brain, along with its precursor glutamine. The glutamate-glutamine cycle maintains an adequate supply of glutamate. Investigating various metabolic patterns may therefore shed the light on neuropathological changes associated with a neurologic disorder. Metabolic changes in the brain are a hallmark of other neurodegenerative disorders with cognitive dysfunction such as frontotemporal dementia (Chawla et al., 2010) and dementia with Lewy bodies (Graff-Radford et al., 2014). In PD, abnormal metabolic ratios of NAA have been observed in the substantia nigra as compared to normal controls (Metarugcheep et al., 2012). Regarding glutamate, one study reports a reduction in the posterior cingulate of non-demented patients (Griffith et al., 2008). In PD-MCI, prior MRS studies have observed reductions in NAA in the occipital lobe (as compared to controls) (Nie et al., 2013) and right PFC (as compared to PD-NC) (Pagonabarraga et al., 2012). In PDD, studies have observed reductions in NAA in the occipital region (Summerfield et al., 2002) and the left hippocampus (Pagonabarraga et al., 2012).

Though Pagonabarraga et al. (2012) correlated prefrontal NAA with frontal subcortical tasks, to our knowledge, no studies have examined glutamate and glutamine in PD patients across the full spectrum of cognitive impairment. Glutamate and glutamine do increase from rest during cognitive tasks (Huang et al., 2015), suggesting that cognitive dysfunction may result from an inability to appropriately increase glutamate and glutamine. Indeed, reduction of glutamate in the PFC occurs in age-related changes in thinking and memory (Grachev et al., 2001) so we hypothesized that PFC glutamate decreases along with cognitive decline. We were also interested as to whether these changes would track changes in other neurometabolites accessed with MRS such as NAA and to provide a more complete metabolic profile. Thus, the aim of this study was to expand on prior research by examining a broader array of metabolic compounds in the PFC using ^1^H-MRS, including for specific neurotransmitters (i) to determine if our cohort of cognitively normal PD patients differ from cognitively normal individuals without PD (CTL) to provide a baseline and confirm/refute previous published studies, and (ii) to determine if varying degrees of cognitive impairment causes metabolic changes in the PFC.

## Methods

### Participants and Cognitive Classification

Participants were recruited from the University of Colorado Hospital Movement Disorders clinics (for the PD groups) as well as fliers posted on campus and signed informed consent to participate in the study approved by the Colorado Multiple Institution Review Board. Inclusion criteria included a diagnosis of probable PD according to the UK Brain Bank Criteria (Hughes et al., 1992). Classification of normal cognition (PD-NC), mild cognitive impairment (PD-MCI), or dementia (PDD), was determined by consensus conference, attended by at least one neurologist (B.M.K., S.K.H.) and one neuropsychologist (M.R.G., L.D.M.) with expertise in PD cognition. Prior to consensus conference meetings, raw scores were transformed to z-scores based on normative data for each of the individual neuropsychological tests, drawn from either testing manuals (Smith, 1973; Benton et al., 1978; Wechsler, 1987; Reitan and Wolfson, 1993; Delis et al., 2000; Schmidt et al., 2005) or additional normative studies (Ruff and Parker, 1993; Tombaugh and Hubley, 1997; Tombaugh et al., 1999; Drane et al., 2002). MDS Task Force Level II diagnostic guidelines for PDD (Emre et al., 2007) and PD-MCI (Litvan et al., 2012) were applied, requiring impairment on at least two tests in one cognitive domain or one test in each of two cognitive domains for classification as cognitively impaired. Of note, we diverged from the MDS recommendations in that we had two neuropsychological tests for all domains tested but one (e.g., visuospatial abilities). Impairment was defined as performance of ≥1.5 standard deviations below age- and education-matched norms (Goldman et al., 2013). PD-MCI was differentiated from PDD based on the results of the neurologist’s functional interview: if significant functional impairment related to cognitive symptoms was present based on clinical impression, a participant was classified as PDD. Exclusion criteria included features suggestive of other neurological disorders and contraindications of MRI, including deep brain stimulation, implanted medical devices, intracranial metal, and motor symptoms expected to interfere with MRI scanning. All study visits were performed in the PD subjects’ best dopaminergic “ON” state. Healthy controls were screened for absence of cognitive impairment (MoCA scores > 26 were included in the study).

### Neuropsychological Battery

PD-NC, PD-MCI, and PDD participants, but not CTL subjects, underwent extensive neuropsychological assessments: Mattis Dementia Rating Scale (DRS-2) (Mattis, 2002); Montreal Cognitive Assessment (MoCA) (Nasreddine et al., 2005); Trail Making Test-A (TMT-A) and Trail Making Test-B (TMT-B) (Army Individual Test Battery, 1944); Symbol Digit Modality Test (SDMT) Oral (Pereira et al., 2015); Delis-Kaplan Executive Function System (DKEFS) Verbal Fluency/Letter Fluency task (Delis et al., 2001); California Verbal Learning Test, Second Edition (CVLT-II) (Fine and Delis, 2011); Boston Naming Test (BNT) (Kaplan et al., 1983); Brief Test of Attention (BTA) (Schretlen, 1997); and Judgment of Line Orientation task (JLO) (Benton et al., 1978).

Cognitive data were checked for missingness and found to violate the assumption that data were missing completely at random (MCAR): Little’s MCAR Test, *p* = 0.007. Therefore, data were imputed to create five multiple imputations. The imputed data did not significantly differ from the original data (all *ps* > 0.05). For data reduction purposes, cognitive composite scores were created using principal components analyses for each set of imputed data separately. Summary statistics for the creation of the composite scores are displayed in Supplementary Table 1. Composites were created from two variables per domain as follows:

- Global cognition: MoCA and DRS-2 total scores
- Attention: TMT-A and BTA
- Language: BNT and verbal fluency
- Learning & memory: CVLT trials 1–5 total score and CVLT long delay free recall
- Executive function: TMT-B and SDMT oral

The Judgment of Line Orientation Test was the only individual test of visuospatial abilities, so a composite score was not created for it.

### MR and MRS Acquisition

**FIGURE 1:**
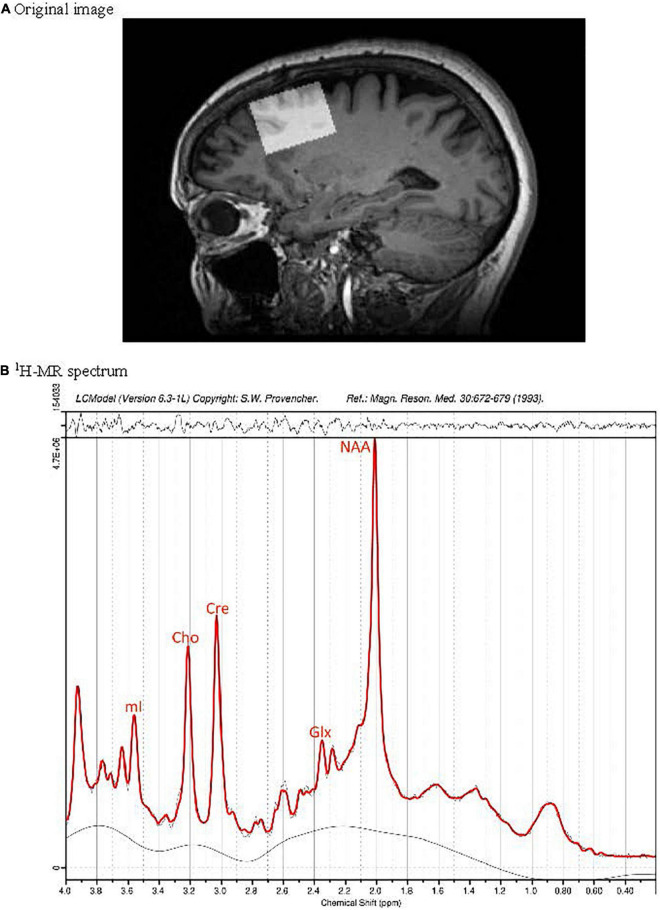
Position of the MRS voxel in the dorsolateral prefrontal cortex of a cognitively normal participant without PD on a sagittal slice of the 3D T1 weighted MRI data set **(A)**. Subplot **(B)** presents the corresponding ^1^H-MR spectrum (black) and fit (red).

MR data were obtained using a Siemens Skyra 3 Tesla wide bore whole-body MRI scanner, equipped with high performance gradient coils (maximum gradient amplitude of 45 mT/m and maximum slew rate of 200 T/m/s) and a 12-channel phased-array head/neck coil. The MRS voxels were placed over PFC (Figure 1A) as described previously (de la Vega et al., 2014) and metabolites were measured using a Siemens’ standard svs-se (PRESS) sequence with parameters TR/TE = 30/2,000 ms, number of averages (NAV) = 128, with out-of-voxel saturation bands placed to minimize artifact from subdural fat. To estimate metabolites ratios in the PFC, LCModel software version 6.3-1L (Provencher, 1993) was used, based on a Bayesian analysis starting with solution spectra basis sets that provide estimates of metabolite ratios without operator bias. Five metabolites were analyzed (Figure 1B): glutamate (Glu), Glx (combined glutamate + glutamine, precursor/product of glutamate), NAA, myoinositol (mI; a glial marker), and total choline (Cho, combined signals from glycerophosphocholine and phosphocholine). Metabolite were expressed as the ratios of their peak areas relative to the peak area of the creatine (Cre) resonance. The averages signal to noise ratio (SNR) and full width half maximum (FWHM) of the spectra in the study (from LCModel output) were, respectively, 49.04 ± 8.9 and 0.049 ± 0.01. Poorly fitted metabolite peaks were excluded from further analysis. The average voxel size was 2.45 ± 0.18 mL.

T1-weighted images were used to determined voxel gray matter (GM), white matter (WM) and cerebrospinal fluid (CSF) fractions in the PFC. CSF, GM and WM of the voxels were segmented *via* calls from Gannet to SPM12 (Functional Imaging Laboratory, The Wellcome Trust Centre for NeuroImaging, in the Institute of Neurology at University College London, United Kingdom). Metabolite ratios were corrected for the voxel CSF content using standardized methods (Chowdhury et al., 2015), since CSF is considered to provide negligible contribution to the Glu and Gln signals.

### Statistical Analysis

All statistical analyses were conducted at *p* < 0.05 for significance using SPSS (IBM Corp. Released 2017. IBM SPSS Statistics for Windows, Version 25.0. Armonk, NY: IBM Corp.). Demographic data were compared between groups by one-way analysis of variance (ANOVA). Chi-squared test was used for categorical data (sex, handedness). CSF-corrected metabolite data were compared between groups by *t*-tests for the PD-NC vs. CTL comparison and one-way analysis of variance (ANOVA) for the PD-NC vs. PD-MCI vs. PDD comparisons. Bonferroni correction for multiple comparisons was used to investigate significant differences. Neuropsychological performance of the PD-NC, PD-MCI, and PDD groups were compared using one-way ANOVA. Bonferroni *post hoc* tests were performed to assess differences between each group. Stepwise linear regression analysis was computed to assess correlations between ^1^H-MRS metabolites and pooled composite scores from the five imputations, as well as scores for the JLO test with age of subjects as a covariate. Two models were run. Corrected Glu/Cre was used as a dependent variable, and age as well as composite score were used as explanatory variables. In addition, a False Discovery Rate correction (Benjamini, 1995) was used to control for multiple comparisons.

## Results

### Demographic/Clinical Characteristics

Data from nineteen CTL subjects, eleven PD-NC participants, twenty-four PD-MCI participants, and eleven PDD participants were used in the ^1^H-MRS study. Participant demographics and neuropsychological performance of the PD group are summarized in Supplementary Table 2 and Table 1, respectively. Groups were not statistically different in terms of demographics or baseline measures with the exception of sex, age, and neuropsychological testing. There was an association between sex and group, χ^2^ (3) = 8.315, *p* = 0.040. Age did not differ significantly between CTL, PD-NC, and PD-MCI participants, but PDD participants were significantly older than the other groups (*F* = 4.498, *p* = 0.006), which is inherently associated with dementia occurrence in PD patients. Therefore, age was used as a covariate for subsequent analyses. Sex was not used as a covariate given that there was no female in one of the PD groups (PDD). Motor severity was also not used as a covariate to avoid multicollinearity issues.

**TABLE 1:** Neuropsychological test scores by cognitive classification for the PD groups.

*Cognitive Domain Test*	CTL	PD-NC	PD-MCI	PDD	*F*-value	*P*-value
***Global Cognition***						
*DRS-2 Total*	N/A	139.55 (4.97), 129–144	134.67 (4.08), 124–143	113.64 (19.56), 82–144	21.423	**0.0000004**
*MoCA*	N/A	27.82 (1.47), 25–30	23.88 (3.47), 16–30	19.55 (5.97), 9–27	12.370	**0.00006**
*Global Cognition Comp. Score*	N/A	0.78 (0.32), 0.14–1.11	0.16 (0.46), −0.86–1.04	−1.12 (1.34), −3.48–0.52	18.796	**0.000001**
***Attention***						
*Trails A*	N/A	−36.64 (15.52), −75.90–(−24.00)	−47.50 (15.16), −76.00–(−27.52)	−109.10 (111.72), −402.00–(−23.00)	5.695	**0.007**
*BTA*	N/A	15.18 (2.71), 9–19	12.70 (4.13), 4–19	10.50 (6.22), 2–20	2.648	0.084
*Attention Comp. Score*	N/A	0.50 (0.50), −0.72–1.09	0.08 (0.64), −1.31–1.04	−0.34 (0.91), −1.21–1.30	3.234	0.051
***Language***						
*BNT*	N/A	56.22 (3.67), 47–59	56.74 (3.03), 49–60	55.17 (3.55), 50–60	0.564	0.574
*Verbal Fluency*	N/A	50.80 (10.16), 36–65	36.46 (14.29), 12–62	26.67 (11.17), 18–48	7.259	**0.002**
*Language Comp. Score*	N/A	0.40 (0.92), −1.65–1.34	−0.02 (0.97), −2.01–1.47	−0.69 (0.99), −1.85–1.03	2.309	0.114
***Learning and Memory***						
*CVLT* (1–5)	N/A	46.18 (7.13), 34–61	33.87 (8.52), 21–52	28.25 (17.86), 11–69	7.759	**0.001**
*CVLT Long Delay Free Recall*	N/A	9.45 (3.01), 4–14	6.78 (3.59), 0–13	3.25 (4.37), 0–12	6.844	**0.003**
*Learning and Memory Comp. Score*	N/A	0.78 (0.66), −0.45–1.99	−0.10 (0.78), −1.52–1.35	−0.79 (1.27), −1.95–2.08	7.889	**0.001**
***Executive Function***						
*Trails B*	N/A	−89.75 (28.61), −159.54–(−66.00)	−138.35 (56.97), −237.64–(−59.00)	−257.00 (173.83), −577.00–(−48.00)	8.784	**0.001**
*SDMT Oral*	N/A	51.45 (8.80), 34–69	37.74 (12.02), 15–59	30.67 (12.52), 19–53	8.103	**0.001**
*Executive Function Comp. Score*	N/A	0.75 (0.48), −0.34–1.57	−0.03 (0.72), −1.21–1.17	−0.51 (1.03), −1.66–1.04	6.875	**0.003**
***Visuospatial***						
*JLO*	N/A	26.18 (3.84), 21–33	25.79 (4.56), 17–34	22.86 (7.01), 12–29	1.179	0.318

*Data presented as mean (SD), range. DRS-2, Dementia Rating Scale; MoCA, Montreal Cognitive Assessment; BTA, Brief Test of Attention; BNT, Boston Naming Test; CVLT, California Verbal Learning Test; SDMT, Symbol Digit Modalities Test; JLO, Judgment of Line Orientation. Means, SDs, f values and p values calculated using SPSS; bold text indicates statistically significant values at p < 0.05.*

**Table:** 

ANOVA		*Post hoc* test (Bonferroni)		
*Cognitive domain*	*F*-value (*p*-value)	*p*-value		
Test		PD-NC:PD-MCI	PD-NC:PDD	PD-MCI:PDD
*Global Cognition*				
DRS-2 Total	21.423 **(0.0000004)**	0.585	**0.0000001**	**0.0000003**
MoCA	12.370 **(0.00006)**	**0.024**	**0.00003**	**0.012**
Global Cognition Comp. Score	18.796 **(0.000001)**	0.082	**0.0000001**	**0.00008**
*Attention*				
Trails A	5.695 **(0.007)**	1.000	**0.014**	**0.013**
BTA	2.648 (0.84)	0.336	0.099	0.773
Attention Comp. Score	3.234 (0.51)	0.288	0.052	0.515
*Language*				
BNT	0.564 (0.574)	1.000	1.000	0.901
Verbal Fluency	7.259 **(0.002)**	**0.017**	**0.003**	0.322
Language Comp. Score	2.309 (0.114)	0.813	0.116	0.419
*Learning and Memory*				
CVLT (1–5)	7.759 **(0.001)**	**0.009**	**0.002**	0.605
CVLT Long Delay Free Recall	6.844 **(0.003)**	0.151	**0.002**	0.066
Learning and Memory Comp. Score	7.889 **(0.001)**	**0.027**	**0.001**	0.174
*Executive Function*				
Trails B	8.784 **(0.001)**	0.370	**0.001**	**0.007**
SDMT Oral	8.103 **(0.001)**	**0.006**	**0.003**	0.543
Executive Function Comp. Score	6.875 **(0.003)**	**0.016**	**0.007**	0.542
*Visuospatial*				
JLO	1.179 (0.318)	1.000	0.495	0.503

*Post hoc group comparisons using Bonferroni correction for each neuropsychological test and composite score.*

### MRS

**FIGURE 2:**
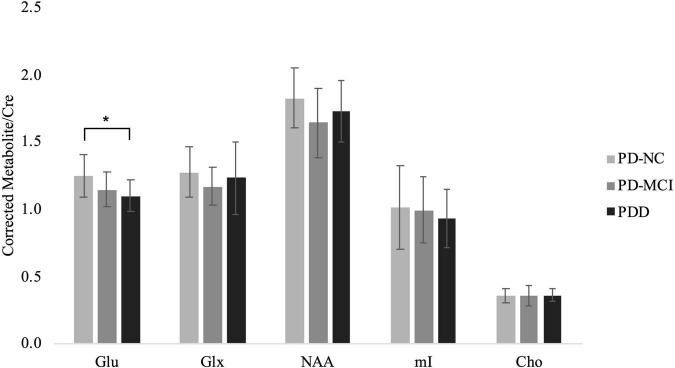
CSF-corrected metabolite ratios to Creatine for the PD groups. Mean ± SD of metabolite ratio to Cre of Glu, Glx, NAA, mI, and Cho in the PD-NC, PD-MCI and PDD groups. Asterisk indicates significant group difference at *p* < 0.05.

CSF-corrected Glx/Cre ratio was increased in the PD-NC compared to the CTL group, however, Bonferroni correction for pairwise comparisons indicated this increase was not statistically significant (Supplementary Table 3). Univariate analysis of CSF-corrected metabolite ratios showed the ratio Glu/Cre [*F*(2,43) = 3.444, *p* = 0.041] in the PFC to be significantly different between the PD groups (Supplementary Table 4). *Post hoc* comparisons showed glutamate to decrease between PD-NC and PD-MCI and also between PD-MCI and PDD groups, but significant differences were only observed between PD-NC and PDD groups (*p* = 0.044; Supplementary Table 4 and Figure 2). Comparisons of NAA, Glx, mI, and Cho ratios among PD groups demonstrated no differences.

### Linear Regression Analysis

**FIGURE 3:**
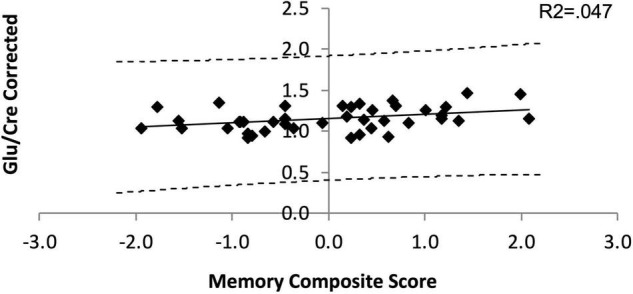
Linear dependance of Glu/Cre on the Memory composite score. The solid line is the line of best fit in the linear regression model. The dashed lines represent the 95% confidence interval (CI) for the upper and lower bounds of the slope parameter.

Averaged Glu/Cre ratios and pooled memory composite scores across PD groups were 0.93 and 1.49 10^–16^, respectively. When controlling for age, the two variables were marginally linearly related (*R*^2^ = 0.047; *p* = 0.062, Supplementary Table 5 and Figure 3) indicating that low PFC glutamate may be associated with poor memory (lower scores). However, given that our *p*-values were above the critical values created by the FDR procedure (Supplementary Table 4) this result was considered as statistically non-significant. No significant linear relationships were observed between Glu/Cre and other neuropsychology composite scores or JLO scores. In addition, no significant linear relationships were observed between any other PFC metabolite ratio and any other neuropsychology composite or JLO scores (Supplementary Table 5).

## Discussion

Our results suggest that before cognitive symptoms appear, older people with or without PD exhibit similar neurometabolic profiles in the PFC. But, when cognitive symptoms have appeared, prefrontal glutamate is decreasing along with cognitive worsening.

Glutamate abnormalities at the subcortical level have been previously noted in animal models of PD (Albin et al., 1989; Greenamyre et al., 1994; Phillips et al., 2006) but not in humans (Clarke et al., 1997; Taylor-Robinson et al., 1999; Clarke and Lowry, 2000; Kickler et al., 2007; Emir et al., 2012). In cortical regions, however, glutamate contents are either unchanged when comparing adults with and without PD (Kickler et al., 2007; O’Gorman Tuura et al., 2018), or decreased in the posterior cingulate cortex when the PD group was selected with a strict non-dementia criteria (Griffith et al., 2008). Despite the exclusion of patients with suspected dementia, dementia with Lewy bodies, or atypical PD, it is unclear if patients with PD-MCI were included. In a neuropathological study, vesicular glutamate transporters VGLUT1 and two immunoreactivity was found significantly decreased in the prefrontal and temporal cortices, suggesting a diminution of Glu neuronal expression (Kashani et al., 2007). These findings and ours indicate that cortical glutamate may be perturbed in a regional manner in people with PD, and that it may be related to their cognitive status. Given an evident link between glutamate receptors activation and memory processes (Riedel et al., 2003), pharmacological studies have started to investigate the use of glutamate ion channels (AMPA receptors) potentiators for enhancing cognition in rodent models of PD (O’Neill et al., 2005). This compound appears to enhance AMPA-mediated responses in cortical and subcortical areas affected by the disease, which is encouraging given the extent of the damage PD causes to the large cortico-basal-thalamo-cortical loops. Further studies are, however, warranted to see if restoring glutamate levels does slow cognitive decline progression in PD.

We also observed that PFC Glu/Cre ratios were marginally associated with memory tasks indicating that worsening memory skills may be associated with decreasing glutamate contents in the PFC. This finding is not surprising given an extensive literature related to glutamate and memory, embracing human and animal models. An animal study at 14.1T showed task- or stimulation-induced increase in glutamate reflects enhanced excitatory neurotransmission as well as increased metabolic activity (Sonnay et al., 2016). Modulation of glutamate measured *via* ^1^H fMRS is therefore a practical method to investigate *in vivo* dynamics of excitatory neural activity driven by task-related cognitive demand. In healthy adults, elevated dorso-lateral PFC glutamate during a working memory task has also been shown *in vivo* (Woodcock et al., 2018). This suggests prefrontal glutamate may be a viable biomarker for memory-related changes, in relationship with diseases affecting cognition or normal aging. Regarding our study, it is possible that investigating glutamate at rest may be subthreshold and therefore not adequately address whether memory impairments are due to dysregulation of local glutamatergic homeostasis.

## Conclusion

In summary, this exploratory study supports the relationship between cognitive dysfunction in PD and metabolite alteration in PFC, suggesting that dysregulation of frontal glutamate may index cognitive changes in PD patients. Limitations of the current study include a relatively small sample size, cross-sectional nature of our dataset, and possibly the lack of activation of glutamatergic networks during rest.

## Data Availability Statement

The raw data supporting the conclusions of this article will be made available by the authors, without undue reservation.

## Ethics Statement

The studies involving human participants were reviewed and approved by the Colorado Multiple Institution Review Board. Inclusion. The patients/participants provided their written informed consent to participate in this study.

## Author Contributions

IB conceptualized and conducted the study, drafted, and reviewed the manuscript. NL-E and FC performed the analyses, drafted, and reviewed the manuscript. MB provided expertise with MRS data acquisition, analyses and interpretation, and reviewed the manuscript. LM helped conducting neuropsychological tests, performed the behavioral analyses, and reviewed the manuscript. EK helped with MRS data analyses and reviewed the manuscript. CM and SR conducted the study and reviewed the manuscript. SH helped with participants’ recruitment and neuropsychological testing, as well as reviewed the manuscript. MG helped with neuropsychological testing battery design and with the consensus on cognitive diagnosis, as well as reviewed the manuscript. BK conceptualized the study and reviewed the manuscript. All authors have reviewed the final version of the manuscript.

## Conflict of Interest

The authors declare that the research was conducted in the absence of any commercial or financial relationships that could be construed as a potential conflict of interest.

## Publisher’s Note

All claims expressed in this article are solely those of the authors and do not necessarily represent those of their affiliated organizations, or those of the publisher, the editors and the reviewers. Any product that may be evaluated in this article, or claim that may be made by its manufacturer, is not guaranteed or endorsed by the publisher.

## Supplementary Material

Supplementary Table 1 Summary statistics for the creation of the composite scores. KMO, Kaiser-Meyer-Olkin Measure of Sampling adequacy; BTS, Bartlett’s Test of Sphericity.

Supplementary Table 2 Participant demographics. Data presented as mean (SD), range or fraction of participants. The high levodopa equivalence standard deviation indicates that the participants were spread out over a wide range of dosages. UPDRS, Unified Parkinson’s Disease Rating Scale. Bold text indicates statistically significant group differences.

Supplementary Table 3 CSF-corrected metabolite measures for CTL and PD-NC. Values are expressed as means ± SD. Using the Bonferroni correction for three comparisons, the *p* value has to be below 0.05/1 = 0.01 for an effect to be significant at the 0.05 level. For these data, all *p* values are far below that, and therefore all pairwise differences are not significant.

Supplementary Table 4 Corrected metabolite measures for the presence of CSF by cognitive classification. Means, SDs and *f*-values calculated using SPSS; *Post hoc* group comparisons using Bonferroni correction for each corrected metabolite level; bold text indicates statistically significant values at *p* < 0.05. Glu, Glutamate; Glx, Glutamate + Glutamine combined; NAA, N-Acetyl aspartate; Cho, Choline.

Supplementary Table 5 Pearson product-moment correlation coefficient related to metabolite measures. Means, SDs, *R*^2^-values and *p*-values calculated using SPSS; * indicates statistically significant values at *p* < 0.05. JLO, Judgment of Line Orientation; B-H, Benjamini-Hochberg. False Discovery Rate correction using the B-H method was used to calculate Critical Values for the Glu/Cre linear regression analyses. *P*-values over the B-H Critical Values are interpreted as statistically non-significant.

## Abbreviations

MoCA: Montreal Cognitive Assessment
SDMT: Symbol Digit Modalities Test
UPDRS: Unified Parkinson’s disease Rating Scale
DRS: Dementia Rating Scale
CVLT: California Verbal Learning Test
Glu: glutamate
Glx: glutamate + glutamine
NAA: N-acetyl-aspartate
mI: myoinositol
Cho: Choline
Cre: creatine
PFC: Prefrontal Cortex
PD-NC: Parkinson’s disease – Normal Cognition
PD-MCI: Parkinson’s disease – Mild Cognitive Impairment
PDD: Parkinson’s disease – Dementia.
