# Supplementary material for: Does Prefrontal Glutamate Index Cognitive Changes in Parkinson’s Disease?
Source: Front Hum Neurosci. 2022 Apr 12;16:809905. doi: 10.3389/fnhum.2022.809905 (PMC9039312; doi:10.3389/fnhum.2022.809905)
Supplement: Supplementary Table 1 — Summary statistics for the creation of the composite scores. KMO, Kaiser-Meyer-Olkin Measure of Sampling adequacy; BTS, Bartlett’s Test of Sphericity. [file Table_1.docx]

| Supplementary Table 1  Summary statistics for the creation of the composite scores | | | | |
| --- | --- | --- | --- | --- |
| Cognitive Domain | KMO | BTS  P value | Eigenvalue total | Cumulative % Variance Explained |
| Global Cognition | .500 | < .001 | 1.773 | 88.665 |
| Attention | .500 | .005 | 1.443 | 72.144 |
| Language | .500 | .005 | 1.447 | 72.346 |
| Learning & Memory | .500 | < .001 | 1.827 | 91.357 |
| Executive Function | .500 | < .001 | 1.748 | 87.377 |

KMO = Kaiser-Meyer-Olkin Measure of Sampling adequacy

BTS = Bartlett’s Test of Sphericity
