# Supplementary material for: Does Prefrontal Glutamate Index Cognitive Changes in Parkinson’s Disease?
Source: Front Hum Neurosci. 2022 Apr 12;16:809905. doi: 10.3389/fnhum.2022.809905 (PMC9039312; doi:10.3389/fnhum.2022.809905)
Supplement: Supplementary Table 2 — Participant demographics. Data presented as mean (SD), range or fraction of participants. The high levodopa equivalence standard deviation indicates that the participants were spread out over a wide range of dosages. UPDRS, Unified Parkinson’s Disease Rating Scale. Bold text indicates statistically significant group differences. [file Table_2.docx]

| Supplementary Table 2  Participant demographics | | | | | | |
| --- | --- | --- | --- | --- | --- | --- |
| Demographics | CTL | PD-NC | PD-MCI | PDD | F/X^2^-value | p-value |
| Sex (Male) | 11/19 | 5/11 | 16/24 | 11/11 | 8.315 | **.040** |
| Age | 66.47 (6.34), 57-80 | 66.91 (7.09), 49-75 | 68.67 (7.70), 57-83 | 76.18 (8.68), 61-92 | 4.498 | **.006** |
| Education (Years) | 16.63 (2.54), 12-22 | 16.45 (3.50), 11-24 | 15.60 (3.12), 7-22 | 16.27 (2.20), 12-20 | .507 | .679 |
| Handedness (Right) | 17/19 | 11/11 | 21/24 | 8/11 | 6.782 | .341 |
| MoCA | 27.89 (1.37), 26-30 | 27.82 (1.47), 25-30 | 23.88 (3.47), 16-30 | 19.55 (5.97), 9-27 | 17.84 | **.00001** |
| Levodopa Equivalence | N/A | 448.18 (566.30), 0-1600 | 499.58 (434.22), 0-1400 | 642.82 (538.08), 100-1540 | .479 | .623 |
| UPDRS Total | N/A | 147.00 (35.96), 81-191 | 137.21 (33.13), 81-190 | 172.91 (19.63), 146-197 | 4.927 | **.012** |
| Hoehn & Yahr | N/A | 2.18 (.72), 1-3 | 2.15 (.63), 1-3 | 2.73 (.72), 2-4 | 3.001 | .060 |

Data presented as mean (SD), range or fraction of participants. The high levodopa equivalence standard deviation indicates that the participants were spread out over a wide range of dosages. UPDRS = Unified Parkinson’s Disease Rating Scale. Bold text indicates statistically significant group differences.
