# Supplementary material for: Does Prefrontal Glutamate Index Cognitive Changes in Parkinson’s Disease?
Source: Front Hum Neurosci. 2022 Apr 12;16:809905. doi: 10.3389/fnhum.2022.809905 (PMC9039312; doi:10.3389/fnhum.2022.809905)
Supplement: Supplementary Table 3 — CSF-corrected metabolite measures for CTL and PD-NC. Values are expressed as means ± SD. Using the Bonferroni correction for three comparisons, the p value has to be below 0.05/1 = 0.01 for an effect to be significant at the 0.05 level. For these data, all p values are far below that, and therefore all pairwise differences are not significant. [file Table_3.docx]

| Supplementary Table 3  Corrected metabolite measures for CTL and PD-NC | | | | |
| --- | --- | --- | --- | --- |
| Metabolite Measures | CTL | PD-NC | *t*-value | P-value |
| Glu/Cre Corrected | 1.13 ± .17 | 1.25 ± .16 | 1.85 | .074 |
| Glx/Cre Corrected | 1.13 ± .17 | 1.27 ± .19 | 2.09 | .045 |
| NAA/Cre Corrected | 1.67 ± .21 | 1.83 ± .22 | 1.87 | .073 |
| mI/Cre Corrected | .88 ± .14 | 1.02 ± .31 | 1.59 | .123 |
| Cho/Cre Corrected | .33 ± .05 | .35 ± .05 | 1.22 | .232 |

Values are expressed as means ± SD. Using the Bonferroni correction for three comparisons, the p value has to be below 0.05/1 = 0.01 for an effect to be significant at the 0.05 level. For these data, all p values are far below that, and therefore all pairwise differences are not significant.
