# Supplementary material for: Does Prefrontal Glutamate Index Cognitive Changes in Parkinson’s Disease?
Source: Front Hum Neurosci. 2022 Apr 12;16:809905. doi: 10.3389/fnhum.2022.809905 (PMC9039312; doi:10.3389/fnhum.2022.809905)
Supplement: Supplementary Table 4 — Corrected metabolite measures for the presence of CSF by cognitive classification. Means, SDs and f-values calculated using SPSS; Post hoc group comparisons using Bonferroni correction for each corrected metabolite level; bold text indicates statistically significant values at p < 0.05. Glu, Glutamate; Glx, Glutamate + Glutamine combined; NAA, N-Acetyl aspartate; Cho, Choline. [file Table_4.docx]

| Supplementary Table 4  Corrected metabolite measures for the presence of CSF by cognitive classification | | | | | | |
| --- | --- | --- | --- | --- | --- | --- |
| Metabolite Measures | PD-NC | PD-MCI | PDD | F-value | P-value |  |
| Glu/Cre Corrected | 1.25 ± .16 | 1.15 ± .13 | 1.10 ± .12 | 3.444 | **.041** |  |
| Glx/Cre Corrected | 1.27 ± .19 | 1.17 ± .14 | 1.23 ± .27 | 1.270 | .291 |  |
| NAA/Cre Corrected | 1.83 ± .22 | 1.64 ± .26 | 1.73 ± .23 | 2.203 | .123 |  |
| mI/Cre Corrected | 1.02 ± .31 | .99 ± .25 | .93 ± .21 | .352 | .705 |  |
| Cho/Cre Corrected | .35 ± .05 | .36 ± .07 | .36 ± .05 | .055 | .947 |  |

Values are expressed as means ± SD.

|  | | | | |  |
| --- | --- | --- | --- | --- | --- |
| ANOVA | | Post hoc test (Bonferroni) | | | |
| Metabolite | *F*-value (*p*-value) | *p*-value | | |  |
|  |  | PD-NC:PD-MCI | PD-NC:PDD | PD-MCI:PDD | |
| Glu/Cre Corrected | 3.444 (**.041)** | .152 | **.044** | 1.000 | |
| Glx/Cre Corrected | 1.270 (.291) | .405 | 1.000 | 1.000 | |
| NAA/Cre Corrected | 2.203 (.123) | .134 | 1.000 | .972 | |
| mI/Cre Corrected | .352 (.705) | 1.000 | 1.000 | 1.000 | |
| Cho/Cre Corrected | .055 (.947) | 1.000 | 1.000 | 1.000 | |

Post-hoc group comparisons using Bonferroni correction for each corrected metabolite level. Glu = Glutamate; Glx = Glutamate + Glutamine combined; NAA = N-Acetyl aspartate; Cho = Choline.
