# Supplementary material for: Does Prefrontal Glutamate Index Cognitive Changes in Parkinson’s Disease?
Source: Front Hum Neurosci. 2022 Apr 12;16:809905. doi: 10.3389/fnhum.2022.809905 (PMC9039312; doi:10.3389/fnhum.2022.809905)
Supplement: Supplementary Table 5 — Pearson product-moment correlation coefficient related to metabolite measures. Means, SDs, R2-values and p-values calculated using SPSS; * indicates statistically significant values at p < 0.05. JLO, Judgment of Line Orientation; B-H, Benjamini-Hochberg. False Discovery Rate correction using the B-H method was used to calculate Critical Values for the Glu/Cre linear regression analyses. P-values over the B-H Critical Values are interpreted as statistically non-significant. [file Table_5.docx]

|  | Supplementary Table 5  Simple stepwise linear regression of corrected and pooled metabolites with cognitive domains in PD groups only | | | | | | |  |
| --- | --- | --- | --- | --- | --- | --- | --- | --- |
|  | | Glu/Cre β Pooled R^2^ value  (P-Value) | Glu/Cre β Pooled  B-H Critical Value | Glx/Cre β Pooled R^2^ value  (P-Value) | NAA/Cre β Pooled R^2^ value  (P-Value) | mI/Cre β Pooled R^2^ value  (P-Value) | Cho/Cre β Pooled R^2^ value  (P-Value) | |
| Global Cognition Composite | | .019 (.404) | 0.008 | .011 (.733) | -.001 (.982) | .010 (.816) | .003 (.776) | |
| Executive Function Composite | | -.007 (.794) | 0.012 | -.044 (.207) | .010 (.831) | -.003 (.946) | -.001 (.893) | |
| Learning & Memory Composite | | .047 (.062) | 0.004 | .024 (.483) | .028 (.575) | .005 (.925) | .0003 (.975) | |
| Language Composite | | -.006 (.826) | 0.017 | -.025 (.486) | -.017 (.786) | -.028 (.579) | -.013 (.179) | |
| Attention Composite | | .003 (.908) | 0.021 | -.006 (.922) | -.0005 (.991) | .004 (.944) | -.001 (.959) | |
| Visuospatial (JLO) | | .0002 (.957) | 0.025 | -.001 (.880) | -.004 (.512) | -.005 (.460) | -.001 (.711) | |

JLO = Judgment of Line Orientation

B-H = Benjamini & Hochberg. False Discovery Rate correction using the B-H method was used to calculate Critical Values for the Glu/Cre linear regression analyses. P-values over the B-H Critical Values are interpreted as statistically non-significant.
